# Time of onset and/or diagnosis of ADHD in European children: a systematic review

**DOI:** 10.1186/s12888-021-03547-x

**Published:** 2021-11-16

**Authors:** Ilaria Rocco, Barbara Corso, Maurizio Bonati, Nadia Minicuci

**Affiliations:** 1 Neuroscience Institute, National Research Council, Padova, Italy; 2 Laboratory for Mother and Child Health, Department of Public Health, Mario Negri Institute for Pharmacological Research, Milan, Italy

**Keywords:** ADHD, Neurodevelopmental disorders, Children, Onset, Diagnosis

## Abstract

**Background:**

Attention-Deficit/ Hyperactivity Disorder (ADHD) is one of the most common childhood neurobehavioral conditions. Symptoms related to this disorder cause a significant impairment in school tasks and in the activities of children’s daily lives; an early diagnosis and appropriate treatment could almost certainly help improve their outcomes.

The current study, part of the Models Of Child Health Appraised (MOCHA) project, aims to explore the age at which children experience the onset or diagnosis of ADHD in European countries.

**Methods:**

A systematic review was done examining the studies reporting the age of onset/diagnosis (AO/AD) of ADHD in European countries (28 European Member States plus 2 European Economic Area countries), published between January 1, 2010 and December 31, 2019. Of the 2276 identified studies, 44 met all the predefined criteria and were included in the review.

**Results:**

The lowest mean AO in the children diagnosed with ADHD alone was 2.25 years and the highest was 7.5 years. It was 15.3 years in the children with ADHD and disruptive behaviour disorder. The mean AD ranges between 6.2 and 18.1 years.

**Conclusions:**

Our findings indicate that there is a wide variability in both the AO and AD of ADHD, and a too large distance between AO and AD. Since studies in the literature suggest that an early identification of ADHD symptoms may facilitate early referral and treatment, it would be important to understand the underlying reasons behind the wide variability found.

**Trial registration:**

PROSPERO registration: CRD42017070631.

**Supplementary Information:**

The online version contains supplementary material available at 10.1186/s12888-021-03547-x.

## Background

Attention-Deficit/Hyperactivity Disorder (ADHD), which is one of the most common childhood neurobehavioral conditions, has been characterized by continually increasing global prevalence rates over the past few decades [1]. A global consensus on the ADHD prevalence rate in children and adolescents has yet to be reached: meta-regression analyses have estimated the worldwide rate at between 5.29% [2] and 7.1% [3], but according to one comprehensive meta-analysis, the best-estimate prevalence rate of study based on case definition was 1.4%, (range: 1.1–3.1) [4].

These conflicting figures have triggered the hypothesis that ADHD is either over diagnosed [5, 6], underdiagnosed, missed, or undertreated [7].

Children with unmanaged ADHD often experience unnecessary impairments and detrimental long-term consequences leading to high personal and societal costs [8, 9]. Early identification and effective management could significantly improve the functioning and overall quality of life of these children and their families.

Healthcare professionals specialized in child psychology, including the American Academy of Paediatrics, advise screening for the disorder early as the preschool period [10] so that those affected can be treated precociously permitting them to achieve their full potential in school and at home [11].

Multiple factors may affect the perception of the disorder by family members and healthcare providers and thus the timing of its diagnosis and treatment [7]. Moreover, there are numerous factors intrinsic to childhood or adolescence that could affect the diagnosis of ADHD including gender, age, race, socioeconomic status, and severity of symptoms [12, 13].

Parents play a central role in recognizing behavioural problems early in their children, their perception, awareness and acceptance of the disease, as their decision to accompany the child to a specialist [14]. Once parents decide to seek help, they need to be able to access specialised care for a timely and accurate diagnosis as well as optimal disease management strategies. Although there is an operationalized psychodynamic diagnostic process, no objective test is at yet available and substantial controversy exists regarding the challenge of formulating a correct diagnosis [15, 16]. In fact, conflicting views continue to exist with regard to the symptoms and psychometric features leading to a diagnosis of ADHD diagnosis [17–19].

Many clinicians depend on and utilize the Diagnostic and Statistical Manual of Mental Disorders (DSM) [20] for guidance in making diagnoses, even if general diagnostic issues (e.g. model of diagnosis and level of impairment) need of better clarification [17] also using different diagnostic criteria, such as the International Classification of Diseases (ICD) and the Research Domain Criteria.

Although the heterogeneity in the methodology of diagnosing of ADHD has resulted in a high variability in prevalence rates around the world [21], differences linked to age at diagnosis (AD) or onset (AO) of ADHD need to be investigated.

Within the Models Of Child Health Appraised (MOCHA) project [22], which has been critically assessing the existing models of primary care for children in 30 European countries (28 European Member States plus 2 European Economic Area countries), Minicuci et al. [23] have been involved in investigating the AO and AD of ADHD.

The current work set out to examine the studies involving children with ADHD in European countries that report their age at onset or diagnosis.

## Methods

Following a systematic review approach and a standardized method of Preferred Reporting Items for Systematic Reviews and Meta-analyses (PRISMA) [24], we searched for studies that reported the AO or AD of ADHD. The review protocol was registered in the PROSPERO database (registration number CRD42017070631). The PRISMA checklist for this systematic review is presented in Additional file 1.

We searched the Medline (PubMed) database for studies in the literature examining ADHD onset or diagnosis published between January 1, 2010 and December 31, 2019. It was decided to limit the review to the last decade because it is the one in which the clinical guideline’s recommendations previously produced by many parties had to be consolidated also with the fifth revision of the *DSM started in 2000 and finished in 2013* [25]. The search terms used were: “ADHD”, “Attention deficit”, “Hyperactivity Disorder” and “Attention disorder” in the title or abstract, combined with “age”, “onset” or “diagnosis” and “child” or “adolescent” in the text word (see Additional file 1 for details). Any study not in English were excluded. In order to include all studies reporting ADHD AO/AD, no exclusion criteria was applied to diagnostic criteria/tools used for participants’ diagnosis in the studies reviewed. The diagnostic criteria or tools adopted in each included study, as well as the inclusion and exclusion criteria followed to select the study sample, have been examined at a later time with other relevant characteristics.

The abstracts of all the articles were read and the full version of the papers for those seemingly fulfilling the selection criteria were retrieved.

Studies were included in this review if they reported the AO or AD for ADHD and were conducted in or referred to data from a European country.

We utilized a standardized form for data extraction that included the following items: the authors’ names, the year of publication, the country in which the study was performed, the journal in which the study was published, the type of study, the aim of the study, the year in which the study was performed, the types of persons composing the study sample (including age and sample size), the diagnostic criteria adopted for the diagnosis, and, of course, the AO/ADs.

Two of the authors (IR and BC) screened all the articles; any differences in viewpoints that arose were resolved through discussion with the third author (NM).

## Results

**Fig. 1:**
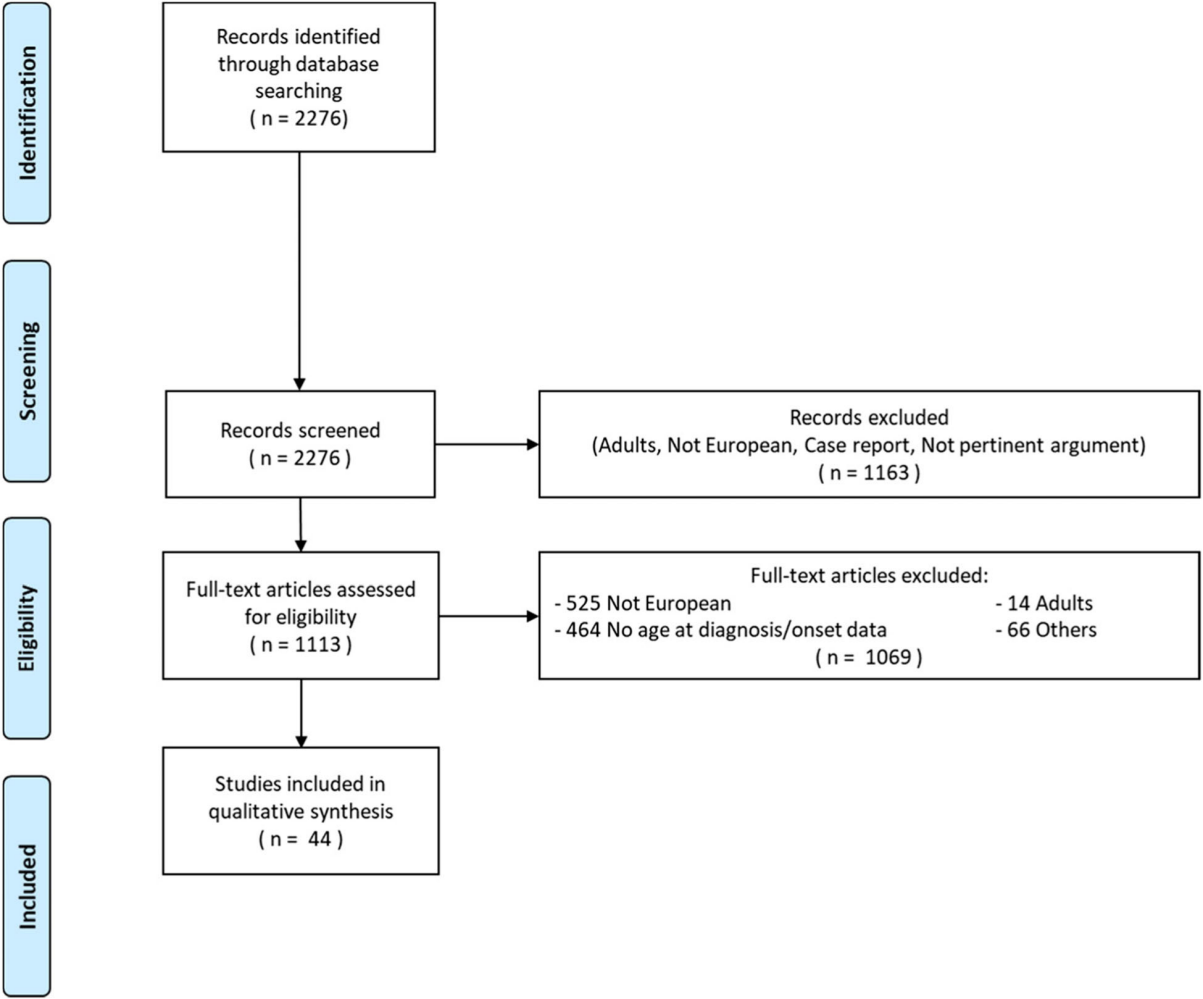
PRISMA flowchart for the selection of eligible studies

The initial PubMed search yielded 2276 studies (Fig. 1). After the abstracts were screened, a total of 1163 articles were excluded, mainly because the population studied and/or the geographic area (did not meet our criteria, in the former case for age, in the latter it referred to studies outside Europe). Out of the 1113 full-text articles reviewed, 49.1% were carried out outside Europe and 43.4% did not report AO or AD. Forty-four articles met our inclusion criteria for this review.

### Study characteristics

The characteristics of the studies included are outlined in Table 1. Twenty-three articles were published in 2010–15 and 21 articles in 2016–19.

**Table 1:** Characteristics of the 44 studies included in qualitative synthesis

Characteristics	n (%)
Year of publication	
2010–2011	7 (15.9)
2012–2013	10 (22.7)
2014–2015	6 (13.7)
2016–2017	11 (25.0)
2018–2019	10 (22.7)
Country	
Germany	5 (11.4)
Sweden	7 (15.9)
Denmark	4 (9.1)
Netherlands	3 (6.8)
Finland	4 (9.1)
France	3 (6.8)
Italy	3 (6.8)
Norway	3 (6.8)
Czech Republic	1 (2.1)
Greece	1 (2.1)
Ireland	1 (2.1)
Spain	1 (2.1)
UK	4 (9.1)
Mix	4 (9.1)
Outcome	
Age at diagnosis	34 (77.3)
Age at onset	9 (20.5)
Both	1 (2.1)
Total	44 (100.0)

One article reported both the AD and the AO, 34 studies reported only the AD, and 9 reported only the AO. The majority of the studies included in this review were conducted in Sweden (7 articles) and in Germany (5 articles), followed by 3 countries publishing 4 articles each.

Table 2 provides a full list of the 44 studies mentioned here, in the order of their publication date; its chronological number is also used throughout the text in all subsequent references to that article.

**Table 2:** List in chronological order of the 44 studies included here

Reference	Country	Type of Study (Year)	Aim	Sample description	Exclusion criteria	Inclusion criteria	Diagnostic criteria and tools	Outcome
Bernardi et al. (2010) [26]	Italy	Cross-sectional (January 2004–January 2008)	To compare bipolar disorder (BD) patients with/without ADHD diagnosis in childhood on clinical and temperamental characteristics.	100 patients with BD, 18 of which with ADHD (10 remitted (aADHD-BD) and 8 persistent (cADHD-BD) in adulthood)	Exclusion criteria were: (1) any clinically significant medical conditions, organic brain disorders, (2) current substance/alcohol abuse or dependence (in the last 6 months), since it may precipitate BD episodes and impair ADHD symptoms at present increasing their intensity; and (3) any lifetime comorbid mental disorders (except previous secondary symptoms of anxiety or substance abuse as based on temporal onset and symptoms severity in remission from at least 6 months), unwillingness or inability to comply with study assessments, or inability to provide informed consent.	Inclusion criteria were: (1) an age range of at least 18 and no greater than 30 years to avoid a retrospective ADHD diagnosis time that was potentially too long; (2) remission of BD for at least 3 months, as assessed by Young Mania Rating Scale and Hamilton Depression Scale scores, in order to reduce possible diagnostic confounders of symptom overlap between ADHD and acute mania; and (3) the presence of at least one parent able to describe the patient’s lifetime course of disease symptoms, in order to confi rm. th e age of onset of ADHD. The presence of at least one parent was considered necessary also to report about the onset and the course of BD symptoms and to distinguish them from symptoms of oppositional defi ant disorder or conduct disorder.	DSMIV-TR (Supported by the WURS)	AO (Mean ± SD, years): -Overall: 6.50 ± 1.04; cADHD: 6.75 ± 0.88; aADHD 6.30 ± 1.15.
Kopp et al. (2010) [27]	Sweden	Descriptive	To clinically describe girls referred for problems with social interaction, attention/academic problems, or tics, comparing symptomatology and comorbidity.	60 clinic girls aged 7 to 16 years with a tested FSIQ ≥80 were selected and matched for age with 60 randomly selected schoolgirls.	Girls with previously diagnosed LD (FSIQ ≤70) had been excluded. However, after full assessment, 12 clinic girls were found to meet criteria for LD (9 of whom were less than age 7 at referral). They were retained because they had not been diagnosed or suspected of LD before entering the study. Other exclusion criteria were defined as parental inadequate command of the Swedish language and serious physical disorders (e.g., cerebral palsy and severe epilepsy).		DSM-IV; Griffiths; WPPSIR; WISC-III; WAIS-R; 10-Item Conners’ Scale; CTRS-R:L; FTF; ASSQ; DSRS; GAF; Severity of Psychosocial Stressors Scale, Children and Adolescents; ADI-R; CAPA; VABS-DLS; ADOS-G	AD (Mean ± SD, years): -Among the clinic girls with ADHD diagnosis (*n* = 46), 13.0 ± 3.4; Among the matched clinic girls with ADHD diagnosis (n = 34):12.7 ± 2.6.
Polanczyk et al. (2010) [28]	UK	Prospective longitudinal (From 1999 to 2000, 7 years follow-up)	To test the implications of extending the ADHD AO criterion from age 7 to 12.	66 met full ADHD criteria; 2 met ADHD criteria, except AO criterion; 1183 without ADHD, symptom onset before age 7 years; 181 without ADHD, symptom onset between ages 7 and 12 years; 547 without ADHD, never had symptoms.			Mother and teacher reports symptoms according to DSM-IV.	At age 12, 66 children (3.3%) met full diagnostic criteria for ADHD, including AO criterion. Among the 181 children with AO between age 7 and 12 years, only 2 met full diagnostic criteria for ADHD (except the AO criterion).
Prihodova et al. (2010) [29]	Czech Republic	Case control (2007)	To evaluate the sleep macrostructure in the ADHD group comparing with controls.	31 patients with ADHD and 26 matched controls (age range 6–12 years).		The enrollment criteria were as follows: (1) ADHD diagnosed on the basis of DSM-IV, (2) no previous pharmacological treatment for ADHD, (3) no history of any chronic physical condition (including obesity), chronic sleep disorder, neurological or other psychiatric disorders (including mental retardation and autism) based on a complete pediatric report and on a neurological and psychiatric examination, (4) no current medication (psychotropic or general) and (5) the patient’s and his/her parents’ willingness to participate in the study and informed consent signed by the parents. All participants were in the prepubertal or early pubertal stages as assessed by Tanner scale. All were of Caucasian origin. They underwent psychological, psychiatric and neurological testing. Information about their sleep habits and sleep disturbances was collected from the parents and children by means of a detailed clinical interview and Pediatric Sleep Questionnaire.	DSM-IV and Children’s Psychiatric Rating Scale; CPRS; CBCL; CMAS; CDI; WISC-III	Parentally reported AO was between 4 and 6 years.
Berek et al. (2011) [30]	Germany	Multicentre, prospective, open-label, single-arm, non-interventional	To compare clinical and health-related quality of life outcomes between children and adolescents treated with Methylphenidate	822 patients with ADHD. Among these, 785 have valid data on age at diagnosis: 565 children (6–12 years) and 220 adolescents (13–18 years).		Children and adolescents aged 6–18 years who had a confirmed diagnosis of ADHD by ICD-10 criteria, and in whom treatment with OROS® MPH was medically indicated and planned by the treating physician, were eligible to participate in the studies.	ICD-10	AD (Mean ± SD/Median/[Range]), years: -Overall: 8.06 ± 2.49/8.0/[1.0–16.0]; −Children: 7.31 ± 1.85/7.0/[2.0–12.0]; −Adolescents: 9.97 ± 2.86/10.0/[1.0–16.0].
Gustafsson and Källén (2011) [31]	Sweden	Population-based (Study initiated in 2005)	To evaluate the impact of pre- and perinatal factors on the risk of developing ADHD.	237 children with ADHD diagnosis and 31,775 typically developing children, born between 1986 and 1996	Individuals for whom no linkage was possible (e.g. children who were born abroad) or children who were born in Sweden but outside Malmo¨ were not included in the final analysis.		DSM-III-R before 1994 and DSM-IV from 1994 onwards	AD varied between 5 and 17 years, with most children diagnosed between 8 and 12 years of age.
Muller et al. (2011) [32]	Belgium, Germany, Ireland, the Netherlands, Spain, Switzerland, UK and Israel	Large multi-centre (recruitment between April 2003 and April 2007)	To analyse the International Multi-centre ADHD Genetics sample with respect to demographic features and psychopathological characteristics.	The sample consisted of 1068 probands with the combined type of ADHD and 1446 ‘unselected’ siblings. Age (Mean ± SD) = 10.8 ± 3.1 years.	Families were excluded from genetic analyses, if either the proband or the participating sibling had an IQ < 70, a diagnosis of schizophrenia or autism, a neurological disorder of the central nervous system or a genetic disorder that might mimic ADHD based on both history and clinical assessment. Children with classical or atypical autism were excluded from the IMAGE project because some genetic regions are known to be associated both with autism and ADHD. There was no rule for assigning proband status to a certain child of a family when several children fulfilled criteria for ADHD-CT.	Recruited families had at least one child with diagnosed or suspected combined type Attention Deficit-Hyperactivity Disorder (ADHD-CT). Further entry criteria for assessment were: white Caucasian ethnicity of all participants, availability of one or more sibling, children between the ages of 5 and 17 years, participation of a minimum of four family members including one parent, and consent of all persons to give blood samples or buccal swabs for DNA extraction.	PACS interview and the DSM-IV items of the CTRS questionnaire	Age at detection of Inattention (I) and Hyperactivity/ Impulsivity (H/I) symptoms (Mean [Range]), years: -Siblings (No diagnosis) I: 6.19 [1–16]; H/I:3.93 [1–15]; −Siblings (Hyperactive/ Impulsive) I: 4.31 [1–11]; H/I: 3.13 [1–6]; −Siblings (Inattentive) I: 4.80 [1–6]; H/I: 3.56 [0–10]; −Siblings (Combined) I: 4.07 [0–10]; H/I: 2.8 [0–7]; −Siblings (All subtypes) I: 4.59 [0–16]; H/I: 3.09 [0–15]; −Probands (Combined) I: 4.22 [0–12]; H/I:2.36 [0–11]. *N.B.: The same data available also by gender*
Durá-Travé et al. (2012) [33]	Spain	Follow-up (Between January and December, 2009)	To determine the repercussions of drug therapy with osmotic-release oral system methylphenidate during 4 years on the weight and height curve of these patients.	187 ADHD patients under treatment with osmotic-release oral system methylphenidate for at least 48 months since their diagnosis. Among them, 158 combined subtype (84.5%) and 29 inattentive subtype (15.5%). Age at baseline (Mean ± SD) = 8.14 ± 1.60 years	The patients who had stopped treatment during school holidays or summer periods were excluded.		DSM-IV	AD (Mean ± SD), years = 8.14 ± 1.60; 84.5% of patients were diagnosed during school age (6–10 years), 10.5% during preschool age (< 6 years), and 5% of patients during adolescence (> 10 years). There were no significant differences in AD regarding sex and clinical subtype.
Garbe et al. (2012) [34]	Germany	Population-Based Cohort (From a first diagnosis of ADHD in 2005, until discontinuation of insurance, death, or December 31, 2008)	To evaluate drug treatment of ADHD in children and youth in Germany with respect to the time until treatment, the initial treatment choice, switches between drugs, and persistence of drug treatment.	6210 children and adolescents 3–17 years of age, with a first diagnosis of ADHD in 2005.They had either received one inpatient or at least two outpatient ICD-10 GM diagnoses within a time interval of 365 days.		Subjects were required to be continuously ensured for at least 12 months before the date of cohort entry.	ICD-10 GM (German modification)	AD (years) M F T 3–5 9.0 7.6 8.7 6–8 36.3 34.9 36.0 9–11 31.3 36.4 32.5 12–14 18.1 14.3 17.2 15–17 5.3 6.8 5.6 Total 100,100,100
Kirov et al. (2012) [35]	Germany		To investigate sleep architecture in children with ADHD by targeting the first-night effect as a possible confounder.	20 unmedicated children with ADHD combined type (8–15 years old; mean 11.24, SD 2.31) and 19 healthy controls, matched for age and gender.	Exclusion criteria for both healthy children and children with ADHD were the presence of internal diseases and neurological problems not associated with ADHD. Subjects with total IQ less than 80 also were excluded. Further exclusion criteria applied for the controls were current sleep problems. Also, none of the controls met any DSM-IV criteria for the presence of any psychiatric disorder. Patients who met DSM-IV criteria for the presence of psychiatric disorders different from ADHD were excluded.		DSM-IV; clinical tests for neurological and internal diseases,including routine electroencephalogram and electrocardiogram	Mean [range] AO = 5.9 [4–8] years
Tuithof et al. (2012) [36]	the Netherlands	Survey (Between November 2007 and July 2009)	To investigate the childhood ADHD association with prevalence and onset of 3 stages of alcohol use (alcohol initiation, regular alcohol use, and alcohol use disorder) and the conduct disorder role in this association.	3309 respondents aged 18–49 years (mean age 32). Childhood ADHD was present in 74 of the respondents.			CIDI (version 3.0) was used according to DSM-IV criteria	The mean AO of ADHD was 6.7 (95% CI: 5.4–8.0) years
Andreou and Trott (2013) [37]	Greece	Matched case-control	To examine the performance of adults, diagnosed with ADHD in childhood, on semantic and phonemic verbal fluency tasks.	30 university students diagnosed with ADHD in childhood (26 combined type and 4 hyperactive impulsive type) and 30 controls. Mean years of age 20.5, SD = 1.5		Students of both groups must have met the following criteria: (a) entered the Greek university through the national Greek system of exams, (b) reported Greek as their native language, (c) were free from medications known to affect the central nervous system, and (d) had no history of neurological or psychiatric disease.Students who were included in the ADHD group must have also met the following criteria: (a) had received an ADHD diagnosis in childhood, according to DSM-IV criteria and (b) obtained a high score in the ADHD questionnaire adapted from Conners’ Hyperactivity Index. Students who were included in the control group must have obtained a low score	DSM-IV;ADHD questionnaire adapted from Conners’ Hyperactivity Index	The mean AD was 6.2 years of age, SD = 0.9.
Bahmanyar et al. (2013) [38]	Sweden	Follow-up (From January 1, 2006 until December 31, 2009)	To describe the paediatric population with ADHD and their pharmacological treatment.	7931 individuals who, for the first time, were diagnosed or medically treated for ADHD before 19 years of age during 2006–2007		All patients who, for the first time, received a diagnosis of ADHD or treatment for ADHD before 19 years of age in Sweden between January 1st, 2006 and December 31st, 2007 were identified using the National Patient Register and the Prescribed Drug Register.	ICD-10, DSM-IV	The mean age of incident paediatric patients with a recorded ADHD diagnosis or treatment for ADHD is 12.0 years (SD = 3.7).
Hodgkins et al. (2013) [39]	France, Germany, Italy, the Netherlands, Spain and UK	Retrospective chart-review (ADHD diagnosis between January 2004 and June 2007 followed up until 2009)	To descriptively illustrate variation in physician practice patterns in the management of ADHD in various Western European countries.	340 physicians reviewed and abstracted charts for 779 patients (130 France, 151 Germany, 144 Italy, 74 the Netherlands, 134 Spain, 146 UK). Each physician managed approximately 20 patients aged 6 to 12 years and 15 patients aged 13 to 17 years.	Patient charts were excluded if there was evidence of enrolment in a randomized clinical trial.	Physicians were screened as eligible for inclusion in the study if they were engaged in clinical practice for between 3 and 30 years, managed the treatment of at least five ADHD patients (aged 6–17 years) per month and were responsible for making ADHD treatment decisions. Physicians were required to identify the most recent ADHD patients (up to a maximum of five patients aged 6–17 years) that they had seen at the time of the review. In order for patients to be included in the study, they should have had a documented diagnosis of ADHD between January 2004 and June 2007 and have had at least 2 years of follow-up post-diagnosis. Patients were also required to have received either pharmacological treatment or BT following the ADHD diagnosis.	DSM-IV; ICD-9/ICD-10; ADHD Connors Test	Mean (SD)/Median/Range AD: -Overall: 8.9 (2.6)/9/ [2–15] years -France: 9.1 (2.5)/9/ [3–14] years -Germany: 8.4 (2.1)/8/ [2–15] years -Italy: 8.7 (2.9)/8/ [4–14] years -the Netherlands: 8.6 (2.6)/9/ [4–15] years -Spain: 9.0 (2.3)/9/ [3–15] years -UK 9.3 (2.8)/9/ [4–15] years
McCarthy et al. (2013) [40]	Ireland	Case control	To explore the resting-state functional connectivity in ADHD and to determine the localization and specificity of ADHD related connectivity differences between adults diagnosed with ADHD in childhood and controls by examining 5 predefined neural networks.	16 adults with combined-type ADHD who underwent careful clinical assessment as children, mean (SD) age at diagnosis: 8.9 (2.1) years; 16 healthy matched controls	Exclusion criteria consisted of previous head injurywith loss of consciousness, comorbid psychiatric disorder or disease, a history of hydrocortisone use, and current alcohol or substance abuse and/or dependency.		Structured Clinical Interview for DSM-IV Axis I Disorders	Mean (SD) AD = 8.9 (2.1) years
Nordström et al. (2013) [41]	Finland	Prospective (From birth, between July 1, 1985 and June 30, 1986, to December 31, 2010)	To complete previous findings about the comorbidity of Disruptive behaviour disorder (DBD) and ADHD and to compare the diagnoses based on a clinical evaluation using K-SADS-PL and the register data.	44 only DBD diagnosis; 91 only ADHD; 72 comorbid DBD and ADHD; 250 no DBD or ADHD. A total of 457 adolescents participated.	Adolescents who were neither ADHD cases nor controls were excluded from analyses.		K-SADS-PL; SWAN scale	The median AO of the psychiatric disorders among adolescents with only DBD was 14.9, IQR (interquartile range) = [6.4–18.9], with only ADHD 7.5, IQR = [4.3–15.1] and with comorbid DBD and ADHD 15.3 (IQR = 8.6–20.3).
Socanski et al. (2013) [42]	Norway	Retrospective chart-review	To investigate the prevalence and characteristics of epilepsy in a large, unselected cohort of children with ADHD.	607 children (82.4% males) aged 6–14 years with ADHD were identified. Of these 14 (2.3%) had a history of epilepsy and 13 of these had active epilepsy	Patients with IQ below50 and those meeting criteria for pervasive developmentaldisorder were excluded		DSM-IV-TRCBCL; CPRS; CTRS; ADHD rating scale IV	Mean (SD) AD of ADHD, years: Total sample 9.4 (2.5); Children with epilepsy 8.2 (2.3); children without epilepsy 9.4 (2.5) (p-value = 0.07).
Caci et al. (2014) [43]	France, Germany, Italy, the Netherlands, Spain and UK	Cross-sectional (From May 2010 to June 2010)	To assess the degree to which ADHD impairs patients’ everyday lives and to identify the areas of life most affected by the condition.	959 children/adolescents aged < 20 were included in the analyses: 535 with ADHD (ADHD group) and 424 without ADHD (control group).	Respondents who provided implausible or impossible answers (e.g. reporting a time to diagnosis that exceeded the age of the child) were excluded, as well as the UK control group (as child age was not collected). Analyses focus on a subgroup of respondents who reported on children/adolescents aged 6 years (respondents with children aged 5 years or younger were excluded).		Caregiver-reported diagnosis	Mean (SD) AD was 7.0 (2.8) years, ranging from 6.3 (2.1) years in Germany to 7.6 (3.1) years in the Netherlands. Diagnosis was obtained following the consultation of 2.7 (2.6) doctors, ranging from 2.3 (1.8) in the Netherlands to 3.2 (4.0) in France, over a mean period of 20.4 (23.9) months, ranging from 12.2 (19.0) in Spain to 31.8 (30.0) in the UK.
Dalsgaard et al. (2014) [44]	Denmark	Prospective population-based (From birth until date of death or December 31, 2010)	To examine whether gender and injuries in early childhood were associated with later being prescribed ADHD medication in 3 groups of patients (with ADHD, ASD, and OPD).	Within the cohort of all persons born in Denmark between 1990 and 2001 (*n* = 852,711), three mutually exclusive groups of patients was identified: 11553 ADHD, 9698 ASD or 48,468 OPD			ICD-10	Age at first psychiatric diagnosis of: - ADHD: Mean (SD) =9.81 (3.85) - ASD: Mean (SD) =8.40 (4.06) - OPD: Mean (SD) =11.40 (5.26)
Genuneit et al. (2014) [45]	Germany	Population-Based prospective birth cohort (From birth in 2000/2001, with follow-up up to age 11)	To investigate the association between Atopic Eczema and ADHD diagnosis, to further determine the temporal sequence, especially with respect to the ages at diagnosis.	770 children. The cumulative incidence of ADHD was 6.2% up to age 11 years (*n* = 48).	We excluded women who left the hospital immediately after birth, gave birth at < 32 gestational weeks, had a child of < 2500 g, or whose infant was transferred to pediatric care after delivery. We also excluded women who were not speaking German, Turkish, or Russian, the languages in which study material and questionnaires were available.		Parental-reported diagnosis and medication	Among the 48 children with ADHD, 21 were diagnosed up to age 8 years and 27 were diagnosed between 9 and 11 years.
Steinhausen and Bisgaard (2014) [46]	Denmark	Representative study based on a large nationwide psychiatric sample (ADHD diagnosis in the years between 1994 and 2010)	To investigate the risk of various medications in comparison to a control group of non-medicated patients with ADHD, and furthermore risk factors including various co-morbid disorders, duration of medication, age at onset of medication, and year of birth for developing SUD.	20,742 ADHD patients aged between 3 and 60 years			ICD-10	The mean AD was 15.20 (SD = 10.08) years.
Sucksdorff et al. (2015) [47]	Finland	Nationwide, nested, case-control (Born between January 1, 1991, and December 31, 2005, followed until December 31, 2011)	To examine the association between gestational age and ADHD by each gestational week. To study the association of weight for gestational age and ADHD.	10,321 children with ADHD were included in the study. Each patient was matched with 4 controls.	Children who had received an ADHD diagnosis before the age of 2 years, but not after that, were excluded. Children diagnosed with severe or profound mental retardation also were excluded. Children for whom information on gestational age or birth weight was not available or clearly inaccurate were excluded.		ICD-9 from 1987 to 1995; ICD-10 since 1996. 88% of subjects met DSM-IV criteria	The mean AD was 7.6 years (SD 2.9 years, range: 3–19 years).
van den Ban et al. (2015) [48]	the Netherlands	Cohort of ADHD patients diagnosed between January 1999 and December 2010	To analyse differences in starting and discontinuation of ADHD medication between native Dutch youth and those with a Moroccan, Turkish or Surinam cultural background with ADHD	817 (11.6% of total patients) patients that had a diagnosis of ADHD. All patients were younger than 19 years at the time of diagnosis. 598 patients were Dutch natives, 143 Moroccans, 52 Turks and 24 of Surinam’s.		younger than 19 years at the time of diagnosis. Had at least 6 months of history in the composed database before the ADHD diagnosis and could be followed for at least 6 months afterwards.	Diagnosis of ADHD at Altrecht (a large institute for mental health care) identified from the Psychiatric Casus Register.	Almost 60% of the patients are diagnosed at the age of 6–11 year old. Mean (SD) age at ADHD diagnosis, years: -Overall: 10.1 (3.5); −Dutch natives: 10.1 (3.5); Moroccans: 9.8 (3.3); Turks: 11.4 (3.8); −Surinam’s: 10.7 (3.7). Total sample age at ADHD diagnosis: 0–5 yrs. 5.6%; 6–11 yrs. 62.8%; 12–18 yrs. 31.6%. Dutch natives age at ADHD diagnosis: 0–5 yrs. 6.2%; 6–11 yrs. 63.0%; 12–18 yrs. 30.8%. Maroccan age at ADHD diagnosis: 0–5 yrs. 4.9%; 6–11 yrs. 67.1%; 12–18 yrs. 28.0%. Turkish age at ADHD diagnosis: 0–5 yrs. 3.8%; 6–11 yrs. 50.0%; 12–18 yrs. 46.2%. Surinam age at ADHD diagnosis: 0–5 yrs. 0.0%; 6–11 yrs. 58.3%; 12–18 yrs. 41.7%.
Caci et al. (2016) [49]	France	Multicentric, cross sectional (Between November 4, 2013 and January 31, 2014)	To describe the health care trajectories in a sample of French children with ADHD	All the 473 patients in the series were under age of 18 (median age was 11.0 years); 382 were boys (81%).	no exclusion criterion was defined	under the age of 18 in whom ADHD diagnosis had been confirmed by the clinician	ADHD diagnosis confirmed by physician	AD: Mean (SD) = 8.07 (2.19); Median = 7.5. AO noticed by caregivers: Mean (SD) = 4.45 (2.25). Age at the first symptoms noticed outside the family: Mean (SD) = 5.00 (2.30).
Lemcke et al. (2016) [50]	Denmark	Longitudinal (from birth until their first ADHD diagnosis or to the end of follow-up on 8 February 2012)	To investigate if children that are later diagnosed with disorders of attention and activity, already early in life have deviations in early development that can differentiate them from children with typical development.	2034 ADHD (F90.0, F90.1, F98.8) cases were included in the study, which corresponds to 2.7% of the study population. 24 children diagnosed with ADHD before 3 years of age were excluded. Mean (SD) [Range] age at end of follow-up, years: ADHD cases 11.4 (1.30) [8.7–13.9]; Study cohort 11.3 (8.6) [13.9–1.35].	children diagnosed with ADHD before 3 years of age were excluded. Indication for treatment was narcolepsy were removed from the cohort.		ICD-10	Mean (SD) [Range] AD, years = 8.4 (1.98) [3.0–13.4]
Rheims et al. (2016) [51]	France	Multicentre prospective observational (Enrolment between November 2011 and September 2014, follow-up 12–16 week)	To investigate the association between the presence of ADHD and the type of epilepsy, the duration of epilepsy, the seizure frequency, the antiepileptic treatments, the co-occurrence of other psychiatric comorbidities.	160 patients aged between 6 and 16 years completed the follow-up, including 58 in whom Methylphenidate (MPH) had been initiated at study entry. 68 children (42.5%) had ADHD-I and 92 (57.5%) had ADHD-C.		(1) age ≥ 6 years and < 16 years; (2) patients having epilepsy according to International League Against Epilepsy (ILAE) classification14 regardless of underlying epilepsy syndrome, seizure frequency, or ongoing antiepileptic drug treatment; (3) diagnosis of ADHD of Inattentive subtype (ADHD-I) or combined Inattentive/Hyperactive- Impulsive subtype (ADHD-C) according to the Diagnostic and Statistical Manual of Mental Disorders, Fourth Edition (DSM-IV) criteria except for the criterion of onset before age of 715; (4) no ongoing specific ADHD treatment, including methylphenidate and atomoxetine.	DSM-IV ADHD-RS	Mean ± SD AO of ADHD symptoms, years: - Overall sample: 5.4 ± 1.9, range= [2–13] - Patients not treated: 5.3 ± 1.8 years - Patients treated with MPH: 5.6 ± 2.1
Sollie and Larsson (2016) [52]	Norway	Follow-up 2007–2008	To examine the associations between child symptoms, demographic variables and the following parent and family characteristics.	Parents of 214 children (mean age at follow-up: 12.6 years, SD = 2.1) with Hyperkinetic disorders recruited from five child and adolescent mental health outpatient clinics.		children with Hyperkinetic disorders	ICD-10 DBRS	Mean age at follow-up: 12.6 years, SD = 2.1. The mean interval from the time of diagnosis to follow-up was 3.7 years (SD = 2.2) and ranged from 1 to 10 years.
van Lieshout et al. (2016) [53]	the Netherlands	Follow-up (Enrolment between 2003 and 2006, follow-up on average 6 years)	To investigate ADHD persistence rates, comorbidity rates, symptom severity, overall functioning and the impact of continued pharmacological treatment.	347 participants with ADHD-combined type aged 5–19 years. Mean age at baseline was 11.4 years (SD = 2.8) and mean age at follow-up was 17.4 (SD = 2.8).		age of 5–19 years, Caucasian descent, IQ ≥70, no diagnosis of autism, epilepsy, general learning difficulties, brain disorders and known genetic disorders. Only participants with a diagnosis of ADHD/C based on the algorithm at baseline were included in the current study.	DSM-IV, DSM-5 CPRS-R:L; CTRS-R:L; CAARS-S:L; SDQ; PACS, symptoms as defined by DSM-IV-TR, K-SADS-PL	Mean AO of the first symptom = 2.25 (SD = 1.52) years.
Abel et al. (2017) [54]	Norway	Large Prospective Cohort of women pregnant in their first trimester from all over Norway during the years 1999 to 2008.	To explore the association between iodine intake from food in pregnancy (as a proxy for long-term iodine intake and status) and (i) risk of specialist-diagnosed ADHD in the child and (ii) maternal report of child ADHD symptoms at eight years of age.	77,164 mother-child pairs were included in this study. ADHD diagnosis was registered in 1725 children (2.2%) by December 2015			ICD-10	The median AD was 8.2 years (IQR: 7.0, 9.5 years).
Bachmann et al. (2017) [55]	Germany	Observational; Nationwide routine data of patients diagnosed between 2009 and 2014	To investigate frequency of diagnosis and treatment for ADHD in children, adolescents, and adults, changing between 2009 and 2014 and transition.	In 2009 there were 214,110 members of the Germany’s largest statutory health insurance company aged between 0 and 69 years (71.4% male, mean age 13.5 [± 31.9] years) with a diagnosis of ADHD; in 2014 there were 274,982 (69.7% male, mean age 14.6 [± 35.1] years).		all insurants with a diagnosis of ADHD who were 15 years old in 2008 and who had been continuously insured until 2014.	ICD-10	Graphical representation of ADHD diagnoses in insureds for 2009 and 2014 by age and sex, based on routine data (administrative prevalence). Modal class: 10–14 years
Balboni et al. (2017) [56]	Italy	A posteriori investigation of information derived from a national database	To investigate which item subsets of the Vineland-II can discriminate children with ADHD or specific learning disorders from peers with typical development.	24 children with ADHD (5–14 years), 61 elementary students with specific learning disorders (6–11 years), and 85 controls with typical development (5–14 years).		Italian native speakers and attended a regular education program. T	DSM-IV-TR diagnosis of ADHD was based on a testing battery assessing attentional and executive functions and on questionnaires given to parents and teachers to evaluate the presence of psychological problems. WISC-III	Children received the diagnosis of ADHD at a mean age of 9 years (range: 5–15 years).
Chen et al. (2017) [57]	Sweden	Cohort (born between 1985 and 2006, followed from their third birthday to 31 December 2009 for ADHD diagnosis)	To estimate the strength and pattern of the familial aggregation of ADHD with greater precision than previously reported.	During the follow-up, 31,865 out of 1,656,943 individuals received ADHD diagnosis			ICD-9 during 1987–1996 and ICD-10 from 1997 onwards; or DSM-IV	Graphical representation of cumulative incidence of ADHD diagnosis among all siblings and all cousins. Median class: 10–15 years
Pohlabeln et al. (2017) [58]	Belgium, Cyprus, Estonia, Germany, Hungary, Italy, Spain and Sweden	Prospective multi-centre cohort	To investigate whether in addition to established early risk factors other, less studied pre-, peri-, and postnatal influences, like gestational hypertension or neonatal respiratory disorders and infections, may increase a child’s risk of developing ADHD.	A total of 15,577 children from 8 European countries were included in the analyses (age range: 2–11.9 years, mean age: 6.2 years, SD: 1.9 years). 192 (15 Belgium, 28 Cyprus, 22 Estonia, 42 Germany, 35 Hungary, 8 Italy, 33 Spain and 9 Sweden) were classified as affected by ADHD.			Parent-reported ADHD diagnosis by a physician or medical health professional	AD: ≤ 4 *n* = 13 (6.8%) (4–6] *n* = 34 (17.7%) (6–8] *n* = 93 (48.4%) ≥ 8 *n* = 52 (27.1%)
Sayal et al. (2017) [59]	Finland	Nationwide population-based	To investigate whether relative age is associated with ADHD diagnosis in a country where prescribing rates are low and whether any such association has changed over time or relates to comorbid disorders.	6136 children born between Jan 1, 1991, and Dec 31, 2004, who were diagnosed with ADHD from age 7 years onwards.	children with severe or profound intellectual disability	children diagnosed with ADHD from age 7 years onwards.	ICD-10	The mean AD in the sample was 9.4 years (standard deviation: 2.4; range: 7–19 years).
Bonati et al. (2018) [60]	Italy	Clinical multicentre; review of patient medical records between September 2011, and December 2017	To confirm the association between relative age (defined as the child’s age within their school year) and ADHD in a different additional national context.	4070 children from age 6 years onwards. 2856 of 4070 subjects evaluated (70%) met the diagnostic criteria for ADHD.	children with severe or profound intellectual disability were excluded	children diagnosed with ADHD from age 6 years onwards.	DSM-IV-TR WISC-III; K-SADS, CBCL, CPRS-R, CTRS-R, CGI-S	The mean AD was 9.3 years (SD 2.5, range 6–17).
Cornu et al. (2018) [61]	France	Double-blind placebo-controlled randomised trial between 2009 and 2011	To investigate the effects of omega-3 supplements in children with ADHD.	162 children aged 6–15 years (Treated *n* = 80; Placebo *n* = 82)	known intolerance to omega-3 fatty acids, intake of fatty acid/fish oil dietary supplements for more than 1 week during the 3 months preceding inclusion, or MPH or other ADHD drug during the month preceding inclusion. Children who required MPH treatment were also excluded to ensure equipoise.	children and adolescents aged 6–15 years referred for hyperactivity symptoms to five reference centres for learning disabilities in France.	DSM-IV-TR CGI-S	Mean (SD) AD: DHA–EPA 7.0 (3.0); Placebo 6.9 (2.9)
Prasad et al. (2018) [62]	UK (England)	Population-based cohort study	To provide estimates of the risk of fractures, thermal injuries, and poisonings in young people with/ without ADHD	15,126 young people with and 263,724 without ADHD		CYP aged 3 to 17 years during the study period of 1998–2012, with at least one diagnosis code or at least one drug code for ADHD in the CPRD, were included in the population of CYP with ADHD.	ICD-10	AD: 3–4 years 7.7% 5–9 years 51.2% 10–14 years 35.4% 15–17 years 5.7%
Dalsgaard et al. (2019) [63]	Denmark	Cohort study included all individuals born from January 1995 through December 2016 and followed up from birth until December 2016	To estimate age- and sex-specific incidence rates and risks of being diagnosed with any mental disorder during childhood and adolescence	99,926 individuals were diagnosed with a mental disorder before 18 years of age. Among these, 30,776 had ADHD diagnosis			ICD-10-DCR	Incidence peaked earlier in boys than girls in ADHD (8 vs 17 years of age)
Granström et al. (2019) [64]	Sweden	Nationwide, population-based cohort study with an observational period from January 1964 to December 2013.	To assess if individuals with Hirschsprung disease have an increased risk for ADHD	739 individuals with HSCR and 7390 controls. Twenty-six of the individuals with HSCR and 202 of the 7390 controls had ADHD.	Exclusion criteria were applied only to HSCR to avoid including neonates with suspected HSCR admitted for rectal suction biopsies where the biopsies turned out to be negative or patients admitted only to a hospital not providing pediatric surgery.		Prescription of any drug used in the treatment of ADHD used as a proxy for the diagnosis.	The mean age at diagnosis of ADHD was not different between the groups, 18.1 years (SD = 8.4) vs 16.7 years (SD = 7.8), *p* = 0.39.
Hoang et al. (2019) [65]	UK	Cross-sectional database study of a national surveillance network of children under 19 years of age between January and December 2016	To describe variations in age of ADHD diagnosis and stimulant prescribing among general practitioner practices in a nationwide network and identify factors that might account for these variations.	3470 children with a coded diagnosis of ADHD		3470 children under 19 years of age with a coded diagnosis of ADHD within the RCGP RSC network were included in the study	Read code	The mean age of first ADHD diagnosis was 10.5 years (95% CI 10.1 to 10.9, median 10, IQR 9.0–11.9)
Root et al. (2019) [66]	UK	Population-based cohort study used electronic record data collected before January 3, 2017, from more than 700 general practices	To estimate the associations with intellectual disability and ADHD and investigate association between relative age and childhood depression.	1,042,106 children aged 4 to 15 years	Children receiving an outcome diagnosis before study entry or with missing sex were excluded.	all children who were registered before January 3, 2017, at a general practice contributing high-quality data to the Clinical Practice Research Datalink (CPRD), and younger than 16 years at the last data collection at that general practice. Children were included from their imputed fourth birthday or from 12 months after registering at a practice contributing research quality data to CPRD, if later.	Read code	Median age at ADHD diagnosis was 8.0 years (IQR, 6.7–9.7)
Sun et al. (2019) [67]	Sweden	Prospective cohort study used national registers to identify individuals born from January 1983, through December 2009	To investigate the all-cause and cause-specific mortality risks in ADHD and to explore the potential role of psychiatric comorbidities	2,675,615 individuals with a mean (SD) age at study entry of 6.4 (5.6) years and a mean (SD) follow-up of 11.1 (3.1) years: 1374790 were male (57,919 with an ADHD diagnosis) and 1,300,825 were female (28,751 with an ADHD diagnosis).		all individuals born in Sweden from January 1, 1983, through December 31, 2009, who were alive and residing in Sweden on their 1-year birthday or January 1, 2001 and followed up until death, emigration from Sweden, or December 31, 2013, with the oldest cohort member censored at 31 years of age.	ICD-10	The mean (SD) AD was 14.3 (5.7) years; 13.5 (5.5) years for male and 16.0 (5.6) for female individuals.
Sourander et al. (2019) [68]	Finland	Population-based case-control study	To investigate the association between maternal cotinine levels during pregnancy and ADHD diagnosis in offspring	1079 patients born between 1998 and 1999 and diagnosed with ADHD and 1079 matched controls			ICD-10	The mean AD was 7.3 years (SD: 1.9; range: 2–13.7 years).
Taylor et al. (2019) [69]	Sweden	Population based twin study focused on all birth cohorts between 1992 and 1999	To investigate the degree to which individuals first receiving community diagnoses of ADHD as adults would display discernible signs of neuropsychiatric impairments as children.	662 individuals with diagnoses of ADHD and 14,474 individuals were the comparison group	All diagnoses were required to have been assigned prior to the age of 18.	All diagnoses were required to have been assigned prior to the age of 18.	ICD-10	74 individuals diagnosed after age 18; 394 diagnosed between the ages of 12–18; 194 diagnosed prior to age 12

Attention-Deficit/Hyperactivity Disorder (ADHD); age of diagnosis (AD); age of onset (AO); ADHD Rating Scale-IV (ADHD-RS); Autism Diagnostic Interview-Revised (ADI-R); Autism Diagnostic Observation Schedule–Generic (ADOS-G); Autism Spectrum Screening Questionnaire (ASSQ); Bipolar Disorder (BD); Birleson Depression Self-Rating Scale (DSRS); Child and Adolescent Psychiatric Assessment (CAPA); Child Behavior Checklist for parents (CBCL); Children’s Depression Inventory (CDI); Children’s Manifest Anxiety Scale (CMAS); Clinical global impressions–severity scale (CGI-S); Composite International Diagnostic Interview (CIDI); Conners’ Adult ADHD Rating Scales-Self-Report: Long Version (CAARS-S:L); Conners’ Parent Rating Scale (CPRS); Conners’ Parent Rating Scale-Revised: Long version (CPRS-R:L); Conners’ Teachers’ Rating Scale–Revised: Long Form (CTRS-R:L); Diagnostic and Statistical Manual of Mental Disorders (DSM); Disruptive Behaviour Disorder (DBD); Disruptive Behaviour Rating Scale (DBRS); Five to Fifteen (FTF) questionnaire; Global Assessment of Functioning Scale (GAF); Hyperactivity/Impulsivity (H/I); International Classification of Diseases (ICD); Methylphenidate (MPH); Parental Account of Childhood Symptoms (PACS); Schedule for Affective Disorders and Schizophrenia for School-Age Children, Present and Lifetime Version (K-SADS-PL); Strengths and Difficulties Questionnaire (SDQ); Strengths and Weaknesses of ADHD Symptoms and Normal Behaviour (SWAN); Vineland Adaptive Behaviour Scales–Daily Living Skills domain (VABS-DLS); Wechsler Adult Intelligence Scale–Revised (WAIS-R); Wechsler Intelligence Scale for Children–Third Edition (WISC-III); Wechsler Preschool & Primary Scale of Intelligence–Revised (WPPSIR); Wender Utah Rating Scale (WURS).

### Diagnostic criteria

In the majority of the articles, the diagnostic criteria used to define ADHD symptoms or to formulate a diagnosis of ADHD was the DSM. One study conducted in Sweden [31] reported that the DSM criteria in the DSM-III-R [70] and in the DSM-IV [71] were used before and after 1994 respectively; while in the study of van Lieshout et al. [53] the DSM-IV and DSM-5 [72] were adopted. In eight papers the 4th edition (DSM-IV) was adopted, in five papers the “text revision” of the DSM-IV, namely the DSM-IV-TR [73], was used. The DSM-IV items of the Conners’ teacher questionnaire were used with the Parental Account of Childhood Symptoms (PACS) interview in the study by Muller and colleagues [32].

The Composite International Diagnostic Interview (CIDI) version 3.0 was used to determine the presence of ADHD according to the DSM-IV criteria in the article by Tuithof and collaborators [36]. Developed by the World Health Organization, the CIDI is a fully structured, lay administered interview used worldwide that has been shown to be a reliable and valid instrument [74].

We also identified 15 papers using the ICD to define ADHD symptoms or to make an ADHD diagnosis. Among these papers, one article [47] used both the 9th [75] and 10th [76] editions; the remaining articles used the 10th edition.

In three articles, multiple sources of information were taken into consideration for the diagnosis of ADHD. The diagnostic criteria of the DSM-IV and the ICD-9/ICD-10 were adopted and the results of Conner’s questionnaire were considered in the six countries involved in the study by Hodgkins and collaborators [39]. In the article by Chen and collaborators [57], the individuals who were diagnosed with hyperkinetic disorder (ICD-9, ICD-10) or ADHD (DSM-IV) were defined as ADHD cases; in Bahmanyar et al. [38] the ICD-10 and DSM-IV were adopted.

The semistructured Schedule for Affective Disorders and Schizophrenia for School-Age Children, Present and Lifetime Version (K-SADS-PL) is a DSM-IV-based diagnostic interview procedure that was used in some of the articles to support ADHD diagnosis [41, 53, 59].

Two studies [65, 66] used Read codes for an ADHD diagnosis. Read codes are clinical terminology developed in the UK by the National Health Service (NHS) based on clinical parameters and usage.

Read codes have become the de facto standard for coding diagnoses, operations, and procedure for all national data sets and statistics on hospital and community health services in the UK.

Lastly, the diagnostic criteria utilized were not specified in six articles. In three papers [43, 45, 58], parents/caregivers of children and adolescents were asked if their children had ever been diagnosed with ADHD by a doctor or other healthcare professional; in a paper by Caci and colleagues [49] the physicians treating children with ADHD were asked to select patients to enrol in the study; finally, the patients were identified from the psychiatric cases and drug registers in two articles [48, 64].

### Age at onset

Eight out of 10 of the studies presenting information on the AO reported the mean, median or age range at symptom onset in the sample of children being studied [26, 29, 35, 36, 41, 49, 51, 53]. The lowest AO was reported by a Dutch study examining a sample made up of 347 patients with combined ADHD, whose ages were between 5 and 19 years; the first ADHD symptom appeared at a mean age of 2.25 years [53]. The highest AO was reported in a study referring to children in Finland: it was 7.5 years in the children with only ADHD diagnosis and 15.3 years in the children with comorbid ADHD and disruptive behaviour disorder (DBD) [41].

The study by Muller and colleagues [32] reported the time of ADHD detection rather than the time of symptom onset which was analysed by comparing probands with combined ADHD with their siblings without ADHD diagnosis or to different subtypes of ADHD.

Polanczyk et al. [28] focused on the implications of extending the ADHD AO criterion from ages 7 to 12 years, since the variation would lead to a negligible increase in ADHD prevalence (0.1% in their cohort) by age 12.

### Age at diagnosis

Thirty-two of the 35 studies presenting information on the AD of ADHD reported its mean, median, range or distribution, one study presented the peak of the ADHD incidence in males and females [63]; in two other studies, information on the AD was inferred through the graphical representations of the cumulative incidence of ADHD diagnosis among siblings and cousins [57] and of the prevalence of ADHD diagnosis in an insured population [55].

The age range in the 24 studies reporting the mean AD was 6.2 to 18.1 years. The lowest mean value, which was reported by Andreou and Trott [37], concerned a group of 30 university students living in Greece who were diagnosed with ADHD during childhood (26 a combined form and 4 a hyperactive impulsive form).

Granström et al. [64] reported the highest mean AD in 26 individuals with Hirschsprung disease and 7390 controls taking any drug for the treatment of ADHD according to the Swedish Prescribed Drug Register.

Two studies reported the mean age for ADHD diagnosis for a specific group of countries [39, 43]. Taking into consideration the same countries (i.e. France, Germany, Italy, the Netherlands, Spain and UK), the two studies identified Germany as the country with the lowest mean age and the UK [39] and the Netherlands [43] as the countries with the highest mean age.

## Discussion

ADHD, one of the most common childhood neurodevelopmental disorders, is characterized by a pattern of inattention and/or impulsivity and hyperactivity, behaviours that can have a dramatic impact on children and on family life [77]. Since early identification of the disease is essential to optimize the quality of life of both the children themselves and their families, there is growing research interest in investigating the timing of diagnosis which can lead to prompt medical attention.

The current study set out to investigate age at the time of onset and/or diagnosis of ADHD in children living in European countries by examining the studies published between January 1, 2010 and December 31, 2019 reporting on the AO and AD of ADHD. The study’s most important finding was that there is a wide variability in both.

Much of the variability could be attributed to discrepancies in study methods. Differences in study designs, ranging from case-control, cohort, to cross-sectional, could have affected the AO/AD as a cohort study could more accurately identify the AO and thus the incidence of ADHD with respect to a cross-sectional retroactive study basing its figures on parent reports.

The studies also show differences in sampling methods. Several studies, in fact, used convenience samples from clinic-based studies; others were based on registry data or medical records. It is reasonable to hypothesise that referred patient samples have lower AO/AD compared to community cohorts given the differences in the severity of the disorder in these populations.

The source of information (self-reported, parent-reported, teacher-reported, doctor-reported) can also significantly influence the figures on the AO/AD registered by the different studies. In a multi-country cross-section study by Caci et al. [43] which assessed the degree to which ADHD impairs patients’ everyday lives, the diagnosis was caregiver-reported and the mean AD was 7.0 years, ranging from 6.3 years in Germany to 7.6 years in the Netherlands. The diagnosis was obtained following the consultation of a mean of 2.7 doctors, ranging from 2.3 in the Netherlands to 3.2 in France, over a mean period of 20.4 months, ranging from 12.2 in Spain to 31.8 in the UK.

The articles by Genuneit et al. [45] and Pohlabeln et al. [58] presented the parent-reported AD: in the first, 44% of the children included in the sample were diagnosed before the age of 8 years and the others between 9 and 11 years; in the second article the percentage of children diagnosed between 9 and 11 years fell to 17%. Although the age range as well as the source of information (parent-reported) of these two articles was the same, the differences in AD could probably be explained by the presence of a comorbidity, that is Atopic Eczema in the case of the first sample.

The presence of comorbidities is an exceedingly important consideration when ADHD diagnosis is being discussed. ADHD symptoms can overlap with those of other disorders, including autism spectrum disorder, disorders of mood and conduct, oppositional defiant disorder, learning difficulties, impaired motor control, poor executive functions (working memory, planning, organisation, and time management), communication difficulties, sleep disorders, tics/Tourette syndrome, epilepsy, and anxiety disorders, that commonly coexist with ADHD.

Socanski et al. [42], who compared the ADs of ADHD in a group of children with epilepsy and a control group, uncovered a statistically significant different in the mean ages: the children with epilepsy had a mean AD of 8.2 years, while those without epilepsy had a mean AD of 9.4 years (*p*-value = 0.07). This result suggests that children with comorbidities related to ADHD have a greater probability of being diagnosed with ADHD at a younger age, presumably because they already have access to some kind of healthcare services and are being monitored by medical specialists.

Thus, the characteristics of enrolled populations (exclusion/inclusion criteria) and the sample size enormously undermine the evaluation and comparison of studies as well as a formal summary of the results. The application of meta-analysis is also impossible due to the lack of publication by all the studies of the absolute values.

The criteria used to diagnose the condition is another important factor that could affect the heterogeneity in the studies considered.

The two main diagnostic systems currently being used are the ICD-10 and the DSM-V [72]. Both systems require that symptoms be present in several settings, for example school/work, home life and leisure activities, and that the onset of symptoms be evident in early life, although this criterion has not yet received a consensus among specialists and has changed over the last decades. For the DSM-V, onset is expected to occur by the age of 12 years; for the ICD-10 and the DSM-IV by the age of 7 years. Since AO itself is one of the criteria of a diagnosis of ADHD in the studies included in our analysis, the AD falls within the ages determined by the diagnostic criteria.

Several research teams have been concerned about the implications of increasing the age of onset to 12 years [78]. Some investigators seem favourable to adopting the new criteria since it has increased the number of ADHD patients receiving help [79]. Other researchers believe that parents’ inability to recall the AO prior to 7 years might give false negative results and reduce some of the diagnostic relevance connected to recalling the AO [80]. A revision of AO criteria should in any case be based on studies assessing the performance of different diagnostic criteria in the population [79].

### Strengths and limitations

To our knowledge, this study represents the first systematic review of the AO and AD of ADHD in European countries. Although we did adhere to PRISMA guidelines [24] to ensure methodological rigour, the study does have a number of potential limitations.

The first is that studies not published in English as well as those not available in PubMed were not taken into consideration.

We would also like to point out that the 44 articles included in this systematic review refer to studies conducted in 13 European countries.

Despite these limitations, and those methodological of analysed studies, the review offers new insights into the timing of the onset and diagnosis of ADHD.

## Conclusions

One of the key functions of primary care is to recognize the symptoms of an illness at an early stage. As far as childhood illnesses are concerned, neurodevelopmental disorders are relatively common and increasing in Europe. Early diagnosis makes it possible to contemplate and implement opportune treatment strategies thus reducing, in this case, some of ADHD’s adverse current and future consequences in the child and family. This study provides a preliminary overview on the timing of the onset and the diagnosis of ADHD in children living in European countries. The long term validity and heterogeneity of the classification systems used to guide diagnoses and the factors behind the social, cultural and genetic differences affecting the timing of identification of the syndrome need further analysis. The fact that Germany has a much earlier AO and AD with respect to the UK and the Netherlands is just one example of differences that need to be clarified. Studies in the literature suggest that identifying ADHD symptoms early on can facilitate early referral and treatment, and thus limit its cost in personal and societal terms [81, 82]. To optimize the quality of the service and of the care delivered is the task of both policymakers and clinical experts. To guarantee an equal standard of care for all children and adolescents with ADHD is a pressing need to reduce the times to complete the diagnostic path, and promptly star with appropriate therapy [83]. However, further studies are necessary to uncover the underlying reasons for the large variability observed in both AO and AD, and reducing the distance between the onset and the diagnosis of ADHD.

## Supplementary Information

**Additional file 1.** PRISMA checklist.

## Data Availability

The data used to support the findings of this study are included within the article.

## Abbreviations

ADHD: Attention-Deficit/Hyperactivity Disorder
AD: Age of diagnosis
AO: Age of onset
ADHD-RS: ADHD Rating Scale-IV
ADI-R: Autism Diagnostic Interview-Revised
ADOS-G: Autism Diagnostic Observation Schedule–Generic
ASSQ: Autism Spectrum Screening Questionnaire
BD: Bipolar Disorder
DSRS: Birleson Depression Self-Rating Scale
CAPA: Child and Adolescent Psychiatric Assessment
CBCL: Child Behavior Checklist for parents
CDI: Children’s Depression Inventory
CMAS: Children’s Manifest Anxiety Scale
CGI-S: Clinical global impressions–severity scale
CIDI: Composite International Diagnostic Interview
CAARS-S:L: Conners’ Adult ADHD Rating Scales-Self-Report: Long Version
CPRS: Conners’ Parent Rating Scale
CPRS-R:L: Conners’ Parent Rating Scale-Revised: Long version
CTRS-R:L: Conners’ Teachers’ Rating Scale–Revised: Long Form
DSM: Diagnostic and Statistical Manual of Mental Disorders
DBD: Disruptive Behaviour Disorder
DBRS: Disruptive Behaviour Rating Scale
FTF: Five to Fifteen questionnaire
GAF: Global Assessment of Functioning Scale
H/I: Hyperactivity/Impulsivity
ICD: International Classification of Diseases
MPH: Methylphenidate
MOCHA: Models Of Child Health Appraised
PACS: Parental account of childhood symptoms
PRISMA: Preferred Reporting Items for Systematic Reviews and Meta-analyses
K-SADS-PL: Schedule for Affective Disorders and Schizophrenia for School-Age Children, Present and Lifetime Version
SDQ: Strengths and Difficulties Questionnaire
SWAN: Strengths and Weaknesses of ADHD Symptoms and Normal Behaviour
VABS-DLS: Vineland Adaptive Behaviour Scales–Daily Living Skills domain
WAIS-R: Wechsler Adult Intelligence Scale–Revised
WISC-III: Wechsler Intelligence Scale for Children–Third Edition
WPPSIR: Wechsler Preschool & Primary Scale of Intelligence–Revised
WURS: Wender Utah Rating Scale
