# Publisher Correction to: Time of onset and/or diagnosis of ADHD in European children: a systematic review

**DOI:** 10.1186/s12888-022-03703-x

**Published:** 2022-01-21

**Authors:** Ilaria Rocco, Barbara Corso, Maurizio Bonati, Nadia Minicuci

**Affiliations:** 1 Neuroscience Institute, National Research Council, Padova, Italy; 2 Laboratory for Mother and Child Health, Department of Public Health, Mario Negri Institute for Pharmacological Research, Milan, Italy

**Correction to: BMC Psychiatry 21, 575 (2021)**

**https://doi.org/10.1186/s12888-021-03547-x**

Following the publication of the original article [1], the authors identified that the corresponding author was incorrectly assigned.

The incorrect corresponding author is: Ilaria Rocco

The correct corresponding author is: Barbara Corso

The original article [1] has been corrected.

## References

[1] Rocco I (2021). Time of onset and/or diagnosis of ADHD in European children: a systematic review. BMC Psychiatry.

